# Unique Presentation of Giant Metastatic Microcystic Serous Adenocarcinoma of the Pancreas

**DOI:** 10.1155/2014/913745

**Published:** 2014-02-19

**Authors:** Cyriac Abby Philips, Chetan Ramesh Kalal, Chhagan Bihari, Amrish Sahney, KN Chandan Kumar, Archana Rastogi

**Affiliations:** ^1^Department of Hepatology, Institute of Liver and Biliary Sciences, D-1, Vasant Kunj, New Delhi 110070, India; ^2^Department of Pathology, Institute of Liver and Biliary Sciences, D-1, Vasant Kunj, New Delhi 110070, India

## Abstract

Tumors of the pancreas that contain substantial cystic components include mainly mucinous cystic neoplasm, intraductal papillary mucinous neoplasm, solid pseudopapillary tumor, and cystadenomas (which encompass microcystic, macrocystic/oligocystic, and rare solid serous adenomas). Microcystic adenoma of the pancreas is a tumor that is benign in nature. Malignant transformation in the tumor with metastases is rare and only about 26 cases have been reported so far. Here we present a giant microcystic adenoma of the pancreas, possibly the largest ever malignant type in this group ever reported in the literature with extensive metastases to the liver and causing extensive compression and encasement on surrounding structures.

## 1. Introduction

Pancreatic cystic tumors fall into the serous or mucinous category. The latter most prominently shows malignant transformation in the long run, while the former is considered benign [1]. Microcystic adenoma of the pancreas is a rare tumor that presents indolently with features of slow growth and abdominal pain secondary to mass effect. These tumors are managed expectantly and surgical management is considered only when the patient becomes symptomatic [2]. The mucinous cystadenomas on the other hand, even though slow growing, have high chances of malignant transformation and hence need to be managed earlier, surgically [3]. Malignant transformation of cystic adenomas of the pancreas was first described in 1989 wherein the tumor showed invasion into surrounding structures and into the liver [4]. Afterwards, about 26 case reports and reviews have been inculcated into the literature on cystadenocarcinomas [5]. Characterization of benign cystic adenomas from cystadenocarcinomas has been extremely difficult. The presence of histological invasion is the only modality that can differentiate these two conditions [6]. One recent case report by Zhu et al. revealed for the first time carcinoma ex microcystic adenoma in which the tumor, without metastases, showed inherent malignant changes [7]. Of all the cystadenocarcinomas reported, our report is unique in three aspects. Firstly, this is the largest cystadenocarcinoma reported in the literature. Secondly, the radiological appearance of the tumor is that of a “parasite mass” that takes up extensive blood supply from the celiac axis, and lastly the tumor extensively involved surrounding structures, notably the biliary tree, producing features of obstructive jaundice, a feature never reported before.

## 2. Case Report

**Figure 1:**
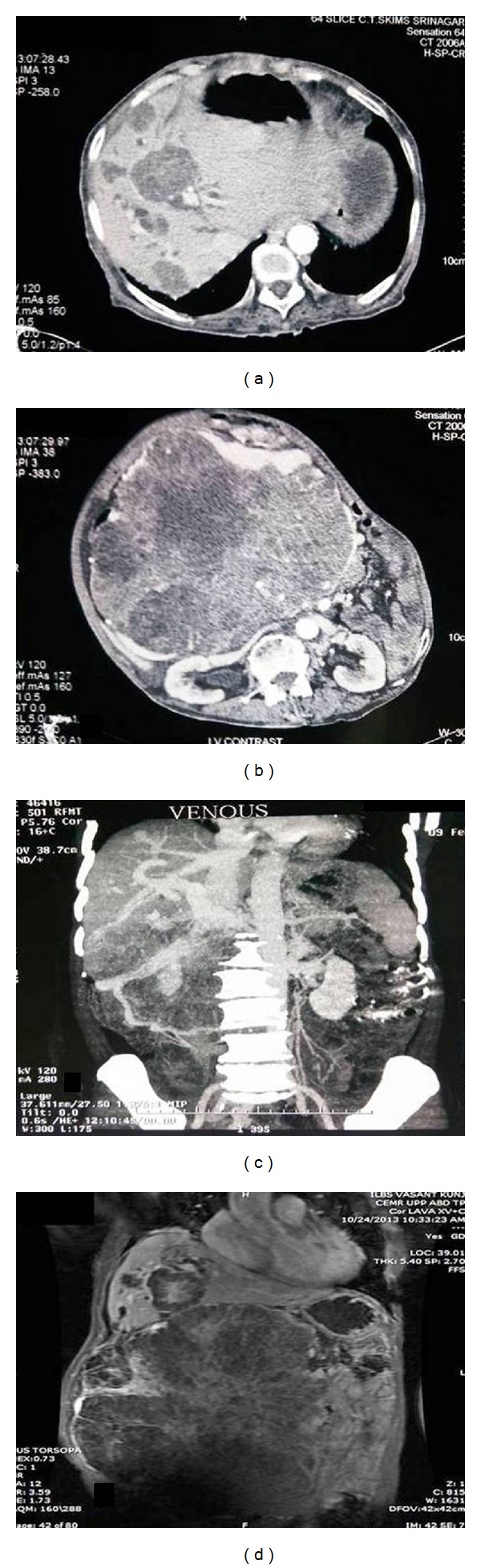
The computed tomographic ((a) to (c)) and magnetic resonance (d) imaging of the abdomen showing features of multiple metastatic lesions in the liver, predominantly in the right lobe (a); 26 cm large heterogenous retroperitoneal mass with areas of necrosis, breakdown, and calcifications involving almost the whole of the abdominal cavity producing mass effect (b); large venovenous communication between the tumor and the major venous channels on venography (c); Large lobulated heterogenous mass with internal septations and surrounding liver metastases extending from the upper to the lower part of the abdomen showing mass effect.

A 70-year-old woman, hailing from Kashmir in India, presented to us with progressively increasing abdominal mass since 11 years. This was associated with progressive appearance of jaundice and dull aching pain in abdomen since 3 months with pruritus for one month that worsened nocturnally. She neither complained of fever, bleeding diathesis, chest pain, cough or palpitations, and altered bowel habits nor did she suffer from anorexia. There was significant unintentional weight loss secondary to early satiety since last one year. There was no history of swelling of legs or puffiness of face during this period. There was no history of swelling over other parts of the body or associated skin changes. The patient is a known case of diabetes mellitus on oral hypoglycemic agents with adequate glycemic control since the last 20 years. Eleven years ago, the patient underwent an open laparotomy for complicated symptomatic gall stone disease and was incidentally detected to have a space occupying lesion of the head of pancreas. The lesion was highly vascular on intraoperative analysis and was left alone at that time with a fine needle aspiration cytology evaluation, which was later confirmed to be that of benign microcystic adenoma. The patient thereafter started noticing progressive enlargement of an epigastric mass which was initially seen 8 years ago. This mass progressed to involve the whole of the abdomen with associated lethargy and early satiety and difficulty in performing routine works. Multiple fine needle aspiration cytology of the mass lesion was done at various hospitals outside, before presentation to our facility. All those reports were noncontributory and confirmed the presence of only blood and cystic elements. On examination, at our center, the patient was found to be conscious and alert but emaciated with temporal hollowing and facial muscle wasting, with good performance status (ECOG-PS 0). Pallor was evident, as were icterus and scratch marks over the abdomen and arms. There was no cyanosis, clubbing, lymphadenopathy, or edema. The abdominal examination revealed the presence of a large epigastric mass, extending towards the right iliac fossa and trespassing on the left lateral aspect of umbilicus with a rough nodular surface, being firm in consistency, nontender, and without visible pulsations. A palpable bruit and a venous hum were also evident. There were areas of engorged, nontortuous veins over the stretched skin surface of the mass. No other organomegaly was appreciable and free fluid in abdomen was difficult to ascertain. The rest of the systemic examination was essentially normal. On further evaluation, the patient was found to have hemoglobin levels of 10 g/dL (normal 12–15) with normal total leukocyte and platelet counts. The ESR was 30 mm in the first hours (normal 0–2). The liver function tests revealed normal bilirubin levels with normal transaminases, in the presence of alkaline phosphatase of 326 IU/L (normal 32–92) and gamma glutamyl transpeptidase of 228 U/L (normal 7–64). The kidney function tests were normal and so were all the viral markers and autoimmune markers. The serum tumor markers, including CA19-9, CEA, AFP, tumoral Beta-HCG, Chromogranin A, and CA-125, were all within normal limits. Computed tomography imaging of the abdomen subsequently followed by angiographic studies in triple phase and magnetic resonance imaging in triple phase revealed the presence of a large lobulated heterogenous mass in the retroperitoneum extending from the undersurface of the liver to the whole of lower abdomen (Figures 1(a), 1(b), 1(c), and 1(d)). The mass measured 26 × 26 cm in size and contained hyperechoic and hypoechoic areas which were hypointense on T1 and hyperintense on T2 weighted images.

**Figure 2:**
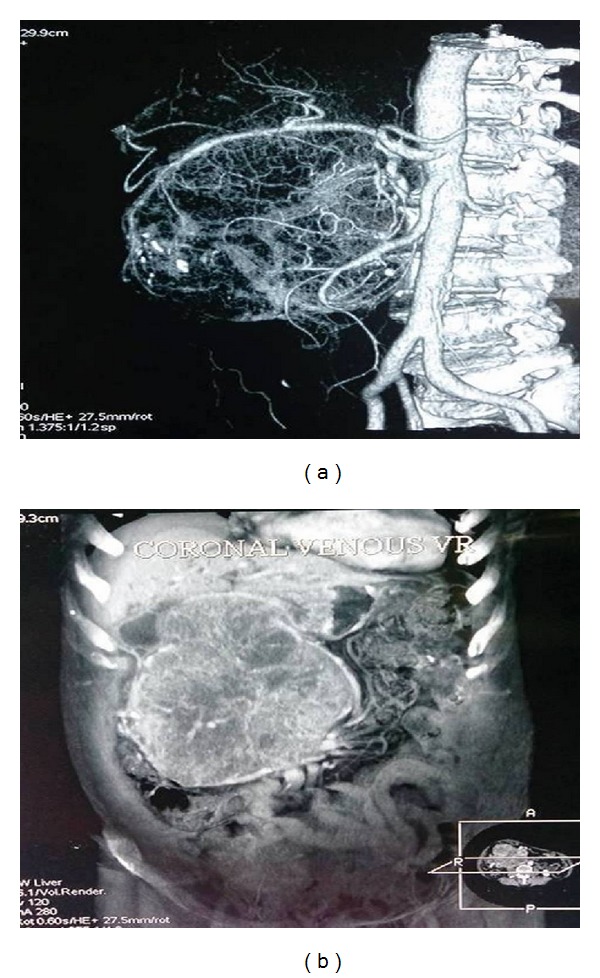
The computed angiographic images of the mass lesion in the abdomen showing features of extensive supply of the tumor derived from the celiac branches (a) and the presence of multiple dilated venous communications within and around the tumoral structure on venography (b).

Multiple hypodense septation (which was T2 hypointense) was noted within the heterogenous mass. Contrast administration revealed moderate heterogenous enhancement with enhancing septation. Compression and displacement of structures at the porta and compression of common bile duct with upstream dilatation of the CBD and bilobar intrahepatic biliary radical dilatation were also evident. The CBD measured 2.7 cm. The inferior vena cava was compressed posteriorly with displacement of the bowel laterally. The tumor derived its supply mostly from the celiac axis with extensive tumoral angiogenesis (Figure 2). Multiple well defined T2 hyperintense and T1 hypointense lesions were noted in both lobes of the liver showing enhancement suggestive of metastases. The liver was otherwise normal and the pancreas could not be delineated. Subsequently the patient underwent a ultrasonography guided percutaneous biopsy from the SOL liver, the histopathological examination of which revealed features of a neoplastic lesion composed of cells arranged in acinar microcystic pattern around the small vessels. These acini and microcysts were lined by single cell layer of uniform cuboidal and Hobnail cells (omit type).

**Figure 3:**
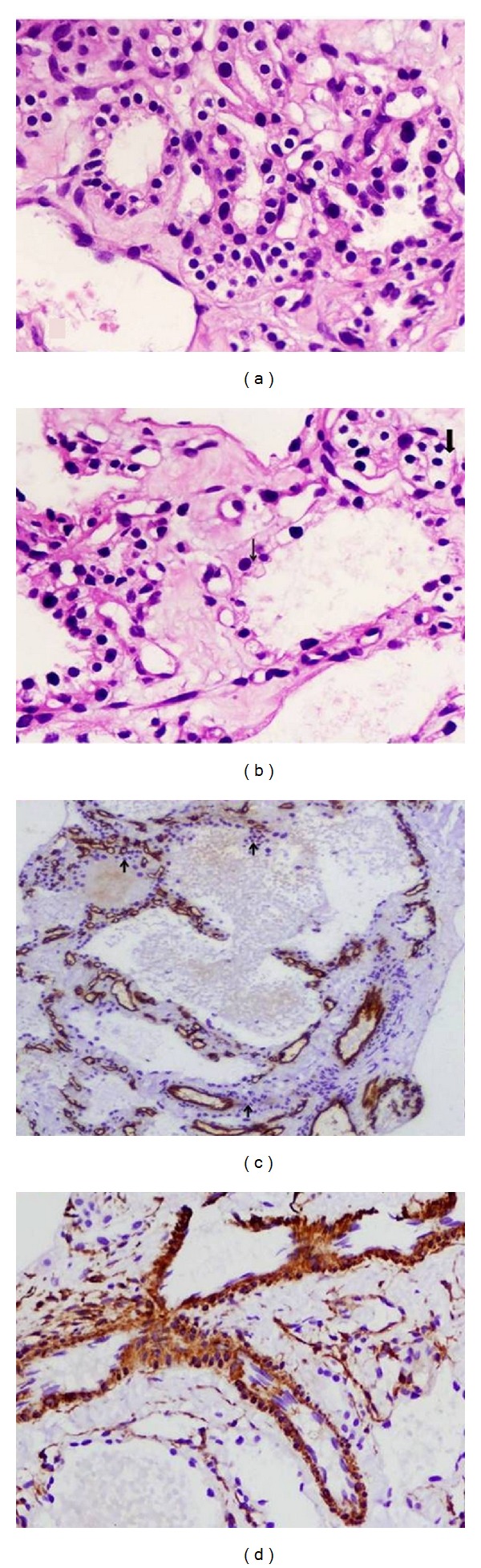
Histopathological evaluation of the SOL from liver. The tumoral mass comprising microcysts lined by cuboidal cells with bland looking nuclei and clear cytoplasm (200x; H&E stain) (a); tumor cells showing clear cytoplasm (thick arrow) and hobnail appearance (thin arrow) (200x; H&E) (b); tumor cells are accompanied by CD34 immunostaining positive vessels (100x) (c); Vessel wall highlighted by smooth muscle actin positivity (200x; SMA immunostaining) (d).

These cells also showed features of monomorphic round hyperchromatic nuclei and moderate amount of clear cytoplasm with inconspicuous nucleoli. The intervening stroma contained several vascular channels with hyalinised stroma. The tumor cells were positive for CK-7, SMA, CD34, and focal EMA. They were negative for focal S-100, CD10, HMB45, MUC1/2, and calretinin (Figures 3(a), 3(b), 3(c), and 3(d)). The glycogen content was documented by PAS positive reaction on histology.

With these clinical, imaging, and histopathological features, a diagnosis of microcystic adenoma was made and the carcinomatous transformation was confirmed by the presence of liver metastases. The patient was not a candidate for surgical or interventional therapy and hence was managed medically for symptom relief. According to the patient's family's wishes, she was then allowed to follow up at her hometown at a nearby center and was lost to follow-up from our side.

## 3. Discussion

Cystadenomas of the pancreas are mainly microcystic, oligo- or macrocystic, and solid serous in type. The cystadenomas are almost always considered benign [7]. The malignant transformation in this group of tumor is documented only in the presence of distant metastases or local invasiveness and histological features suggestive of vascular/lymphatic or neural invasion. The prevalence of carcinomatous change in microcystic adenomas has been reported to be 3% [8]. Since its first description in 1989, the few cases reported relied mostly on histological proof rather than clinical or radiological imaging. The average age range at presentation is 66 to 70 years and more than 50% of the affected patients are female [9]. The commonest presenting complaint is abdominal pain and the least common includes that of jaundice and palpable mass [10]. Our case report showcases a presentation not reported before, that of surgical obstructive jaundice and abdominal pain with a large palpable mass per abdomen. These tumors show synchronous (as in our case) or metachronous liver metastases. There have also been case reports where lymph nodal, splenic, neural, perineural, stomach, lung, adrenal, peritoneal, and mesenteric invasions were also documented [11]. Our case report is the first one to document involvement of the biliary tree with presentation of surgical obstructive jaundice and extensive vascular compression. Cystic tumors of the pancreas have varied behavior and histological characteristics. The serous cystadenoma (also known as microcystic adenoma of the pancreas, unless otherwise specified) is a benign tumor that is usually asymptomatic and is diagnosed incidentally during work up for other conditions. In our case, the patient underwent cholecystectomy many years before this presentation and was incidentally detected to have a mass in the head of the pancreas, which, at the time, was diagnosed as microcystic adenoma, and further management was abandoned in view of benign nature and absence of symptoms. Follow-up was suggested, but the patient sought follow-up medical help only when the tumor was very large and surgical intervention was not feasible. The conclusion that surgical treatment in management of this tumor should be commenced only when the patient is symptomatic should be changed; in lieu with case reports, the malignant potential of this tumor group is getting more realized and patients, even if they are only a few, should be given the benefit of cure eventually, even though mean survival is about 36 months even with metastatic disease [1, 2]. Surgical management can be extended to a selected group of patients in whom the follow-up shows considerable growth and a change in clinical behavior. Microcystic adenomas are found throughout the pancreas with around 50% located in the head of the pancreas. These tumors have an average size of 6 cm, are well demarcated, and consist of multiple small thin walled cysts that contain straw colored fluid. In cases that reported malignant transformation, the average size ranged from 10 to 14 cm [12]. Our case was unique in that aspect. The tumor we report here is massive, being 26 cm in size, one that filled almost the whole of abdomen with extensive compression features on surrounding tissues. On computed tomography, microcystic adenomas appear well circumscribed with innumerable cysts. Sometimes, the tumor shows features of calcifications within the lesion or in the capsule and a central scar with a starburst pattern which is seen in about 30% of the cases [13]. Another unique presentation in this case was that of a huge retroperitoneal mass with solid and cystic heterogeneous components with patchy arterial enhancement with biliary tree involvement and vascular and surrounding tissue compression with angiographic findings suggestive of a “parasite” like mass lesion taking on extensive arterial supply from the celiac axis, a feature never presented in cases before. The histological findings of microcystic adenoma include the presence of single layer of small epithelial cysts lined by cuboidal cells with round to oval nuclei and pale to clear cytoplasm. Nuclear atypia is absent and mitoses are very rare. The cytoplasm is clear to pale and contains glycogen which stains positive for PAS as noticed in our case. Benign and malignant variants have identical histological features. The only distinguishing feature is presence of gross or microscopic evidence of invasiveness [14]. Immunohistochemistry normally reveals positivity for CK7, CK8, CK18, CK19, EMA, alpha-inhibin, and NSE.

Calretinin, CEA, chromogranin, MUC1, MUC2, S-100, HMB-45, CD-31, and vimentin are usually negative [15]. In our patient, the staining pattern was consistent with that of microcystic adenoma. The invasiveness was evident grossly and also microscopically with CD34 staining being positive, that is specific to vascular involvement. Treatment options in early tumor have been gladdening, but in late stages, with the development of metastases with large growth, surgical options they become moribund. Considering the age of the patient and also the associated comorbid conditions, surgical or ablative management was not provided. In conclusion, microcystic adenomas are rare tumors of the pancreas that cannot be completely judged to be benign in nature. They present variedly in patients and the choice of early surgical management should be given to the patient in the event of clinical and biological variation during follow-up which is picked up by the treating physician/surgeon.
